# A Metabonomic Study of the Effect of Methanol Extract of Ginger on Raji Cells Using ^1^HNMR Spectroscopy

**DOI:** 10.1155/2014/572534

**Published:** 2014-12-28

**Authors:** N. Parvizzadeh, S. Sadeghi, S. Irani, A. Iravani, Z. Kalayee, N. A. Rahimi, M. Azadi, Z. Zamani

**Affiliations:** ^1^Department of Biology, Islamic Azad University, Science and Research Branch, Tehran 1477893855, Iran; ^2^Biochemistry Department, Pasteur Institute of Iran, Pasteur Avenue, Tehran 1316943551, Iran

## Abstract

Cancer is currently a major international health problem. The development of resistance to chemotherapy has resulted in the search for herbal drugs. Ginger is a medicinal plant with several clinical applications. Metabolomics is a simultaneous detection of all the metabolites by use of ^1^HNMR or mass spectroscopy and interpretation by modeling software. The purpose of this study was to detect the altered metabolites of Raji cells in the presence of ginger extract* in vitro.* Cells were cultured in the presence and absence of methanolic ginger extract in RPMI medium. IC_50_ determined by MTT and lipophilic and hydrophilic extracts were prepared from control and treated groups which were analyzed by ^1^HNMR. The IC_50_ was 1000 *μ*g/mL. Modeling of spectra was carried out on the two groups using OSC-PLS with MATLAB software and the main metabolites detected. Further analysis was carried out using MetaboAnalyst database. The main metabolic pathways affected by the ginger extract were detected. Ginger extract was seen to effect the protein biosynthesis, amino acid, and carbohydrate metabolism and had a strong cytotoxic effect on Raji cells* in vitro.*

## 1. Introduction

According to GLOBOCAN (Cancer Incidence and Mortality Worldwide) 2012, an estimated 14.1 million new cancer cases and 8.2 million cancer-related deaths occurred in 2012, compared with 12.7 million and 7.6 million, respectively, in 2008. Prevalence estimates for 2012 show that there were 32.6 million people (over the age of 15 years) alive who had had a cancer diagnosed in the previous five years. Projections based on the GLOBOCAN 2012 estimates predict a substantive increase to 19.3 million new cancer cases per year by 2025, due to growth and ageing of the global population. More than half of all cancers (56.8%) and cancer deaths (64.9%) in 2012 occurred in less developed regions of the world [1].

Cancer is treated with surgery, radiotherapy, chemotherapy, hormone therapy, biological therapy, and targeted therapy [2]. Current anticancer drugs used in chemotherapy are usually immunosuppressive and cause severe side effects [3], some of them resulting in drug resistance which is a major problem in cancer treatment process [4]. Studies have shown that plant sources of anticancer agents and their derivatives are very useful for the treatment or prevention of cancer in humans. These herbal compounds are important origins of clinically valuable anticancer agents. For example, they include alkaloids from the Madagascar periwinkle such as vinblastine which is used to treat Hodgkin's lymphoma and vincristine for non-Hodgkin's lymphoma and also the camptothecin derivatives from the bark and stem of* Camptotheca acuminata*, topotecan and irinotecan, used for lung and ovarian cancer [5].

Ginger has been mentioned in traditional medicine in China and India and has been used for more than twenty-five centuries [6]. It is recorded as a food supplement in the list of safe plants of FDA [7]. WHO has recognized ginger as a useful medicinal herb [8]. Several studies indicate the effect of this plant for treatment of different types of cancer cells including lung, ovarian, colon, breast, skin, prostate, and pancreatic cancers [9]. Ginger contains active ingredients; the most important ones from the lipophilic extracts of its rhizome include gingerols, which can convert shogaol and zingerone to 4-paradol. The particular flavour of ginger depends on 6-gingerol and along with 6-shogaol is responsible for its medicinal activities including antipyretic, analgesic, antitussive, and antihypertensive effects as well as antioxidant, anticancer, anti-inflammatory, antiangiogenesis, and antiarteriosclerosis properties [10].

Metabonomics is a novel field dealing with simultaneous study of the entire metabolites in the body, resulting in the metabolic model of the cell using high throughput technology like ^1^HNMR and LC-MS followed by advanced software and multivariate analysis methods. Metabonomics is widely used in toxicology and drug testing and has been used to study the difference between diseased and normal states in various body fluids, tissues, microbes, parasites, and cell lines. Its use in diagnosis of different ailments like arthrosclerosis, diabetes, and certain cancers is being studied [11].

The cell lines used in this study are Raji cells which are cultured from a line of lymphoblastoid cells derived from a human Burkitt (non-Hodgkin) lymphoma which is a cancer of the lymphatic system, particularly B lymphocytes found in the germinal center. These cells besides being used as a transfection host help understand the hematopoietic and other cell malignancies [12]. We have studied the effect of methanolic ginger extract on the metabolites of Raji cells* in vitro* using ^1^HNMR spectroscopy.

## 2. Materials and Methods

### 2.1. Preparation of Ginger

Ground ginger powder was obtained from dried ginger from Mumbai, India. Its methanolic extract was purified using a Soxhlet extractor. In brief, 30 grams of ginger powder was loaded into the cartridge, 300 mL methanol was added, and extraction was started. The resulting extract was purified by the condenser and vacuum pump method. 0.7 grams of sticky extract was dissolved in 7 mL 0.01% DMSO (dimethyl sulfoxide) which gave 100 mg/mL. This was named as ginger stock.

### 2.2. Cell Culture

The Raji cell line (B-cell lymphoma) was supplied by the Pasteur Institute of Iran. It was cultured in a medium containing RPMI-1640 and 10% fetal calf serum, penicillin, and streptomycin. The cells were then incubated at 37°C with 5% CO_2_ at 95% humidity.

### 2.3. Treatment of Raji Cells with Ginger

Three 12-well plates were selected. To each well, 1 mL of culture medium and 1.6 × 10^4^ Raji cells were added and treated with different concentrations of ginger 0.1%, 0.01%, and 0.001% of stock for 24, 48, and 72 hours. DMSO-solved ginger extract was diluted and added to 12-well plates in different concentrations. The test was carried out in duplicate. Control samples lacking the extract were cultured in each plate in duplicate.

### 2.4. Viability Test

Cells were counted for viability using trypan blue method at 24, 48, and 72 hours [13].

### 2.5. MTT Assay

MTT [3-(4,5-dimethylthiazol-2-yl)-2,5-diphenyltetrazolium bromide] assay was also carried out at the same time points.

Briefly, 10,000 cells were placed in 96-well plates and ginger extract was added for 48 hours. The culture supernatant was then discarded, and the cells were incubated with 50 *μ*g/mL MTT stock solution in PBS for 3 to 4 hours at 37°C. After adding 100 *μ*L Formazan in methyl sulfoxide and shaking for 30 min the absorbance was read at 570 nm using Elisa-Reader instrument [14].

### 2.6. Cell Extraction

Methanol-chloroform-water extraction was performed as previously described [15]. The extraction procedure was performed on a crushed ice bath at 4°C. Briefly, cell pellets were resuspended in 500 *μ*L of ice-cold 2 : 1 (v/v) methanol : chloroform solution and then transferred into a 1.5 mL Eppendorf tube. After vortexing, the tubes were incubated on a mixer for 10 min at 4°C. Then, 250 *μ*L of ice-cold H_2_O 1 : 1 (v/v) chloroform/H20 was added and mixed using a vortex mixer. The tubes were sonicated on ice for 10 min and centrifuged for 5 min at 18000 ×g. The top hydrophilic and the bottom lipophilic extract were separated into different Eppendorf tubes. Water was removed from the sample by lyophilization, as presence of water will result in an additional peak in the ^1^HNMR spectrum and cause interference in the spectra. The samples were lyophilized and stored at −20°C until analysis.

### 2.7. Preparation for ^1^HNMR

Lyophilized hydrophilic cell extracts were resuspended in 200 *μ*L of buffer (150 mM potassium phosphate at pH 7.4, 1 mM NaN_3_, and 0.01% and trimethylsilyl propionate (TSP) in 100% D_2_O (deuterium oxide: the required quantity of buffer for each sample was originally prepared in H_2_O, lyophilized, and reconstituted in 100% D_2_O)), and the lipophilic cell extracts were resuspended in 200 *μ*L deuterated chloroform. Both the extracts were analyzed by ^1^HNMR analysis [15].

### 2.8. ^1^HNMR Spectroscopy

The cell suspensions were placed in 5 mm probes (Bruker), for analysis. All ^1^HNMR spectra were recorded on a Bruker spectrometer operating at 400 MHZ spectroscopy by method 1D 1H CPMG (Carr-Purcell-Meiboom-Gill) spin-echo NMR. The temperature of the sample was maintained at 298 K. For each sample 128 transients and 16 dummy scans were collected into 32 k data points of 45 min. The experiments were performed with a spectral width of 5200 HZ, acquisition time of 3.15 s, and relaxation delay of 1.5 s [15].

The ^1^HNMR spectrum includes information about the effective metabolites present in the treated and control groups (both lipophilic and hydrophilic extracts of each). The spectra were then analyzed by MestReC Nova software. Correction of baseline was polynomial correction and automatic phase correction and chemical shifts were referenced to external 0.1% TSP in D_2_O. All spectra were binned into 1000 parts and their normal intensity and chemical shift were entered into Excel file.

## 3. Data Processing

### 3.1. Chemometrics Analysis

The Excel files were entered into MATLAB 6.5 and PLS was implemented with the PLS-Toolbox version 3.0 analyzed by multivariate analysis methods using orthogonal signal correction and Partial linear square (OSC-PLS) [16].

### 3.2. Identification of Metabolites

Metabolites corresponding to these resonances were then identified using chemical shift assignments of spectra of metabolites of the cell extracts based on comparison with chemical shifts of metabolites in Human Metabolome Database Data Bank (HMDB) (http://www.hmdb.ca/metabolites) and in other published data. Analysis of metabolites was carried out using MetaboAnalyst software (http://www.metaboanalyst.ca/).

### 3.3. Statistical Analysis

Statistical analysis was carried out by SPSS version 19. The results are presented as mean ± SEM. Analysis of variations was done and comparisons between study groups were performed with ANOVA and Student's *t*-test. Differences were considered significant at *P* < 0.05.

## 4. Results

**Figure 1:**
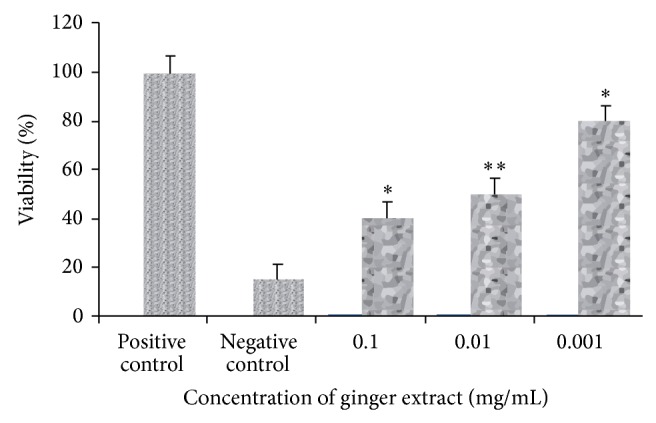
MTT assay showing effect of ginger extract on Raji cells. IC_50_ obtained at 0.01% dilution of stock at *P* < 0.05.

The 48 h effect of alcoholic ginger extract on Raji cells and percent live cells are shown in Figure 1. IC_50_ of Raji cells is seen at 0.01% dilution of stock which is 1000 *μ*g/mL.

**Figure 2:**
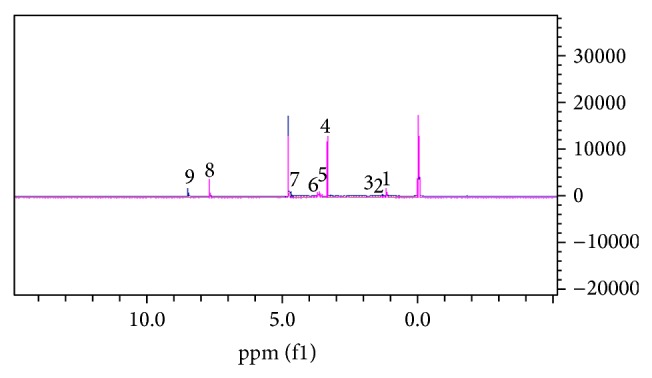
Superimposed spectra of hydrophilic phase of control and experimental Raji cells exposed to methanolic ginger extract. The greatest changes observed were in the 3.0-4.0 chemical shift. Metabolites are (1) isobutyryl-L-carnitine, (2) isoleucine, (3) homo-L-arginine, (4) D-mannose, (5) fructose-6-phosphate, (6) glucose, (7) S-adenosylhomocysteine, (8) 5-methylcytidine, and (9) NADP.

**Figure 3:**
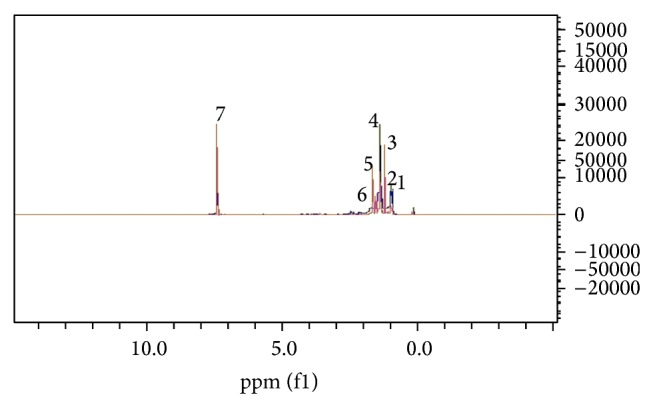
Superimposed spectra of lipophilic phase of control and experimental Raji cells exposed to methanolic ginger extract. Changes were observed in the 1.0-2.0 chemical shift range. Metabolites are (1) 2-ketobuytric acid, (2) isobutyryl-L-carnitine, (3) isoleucine, (4) lactate, (5) homo-L-arginine, (6) lysine, and (7) CDCL3.

The superimposed spectra of the hydrophilic phases of control and Raji cells exposed to methanolic ginger are seen in Figure 2. The greatest changes are seen in the 3.0-4.0 chemical shift. The superimposed spectra of lipophilic extract of control and Raji cells exposed to methanolic ginger extract and the greatest changes are seen in the 1.02.0 chemical shift areas (Figure 3). After this, the Excel files of normal intensity of the spectra were entered into MATLAB and OSC-PLS modelling was carried out in which only 1 orthogonal signal was removed.

**Figure 4:**
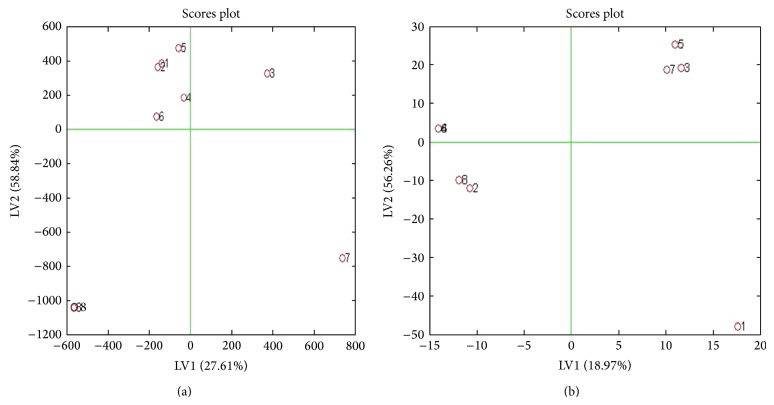
(a) Score plot of OSC-PLS of control and ginger treated hydrophilic phase extract. (b) Score plot of OSC-PLS of control and ginger treated lipophilic phase metabolites. In both figures odd numbers indicate those treated with drug samples, and even numbers indicate control.

**Figure 5:**
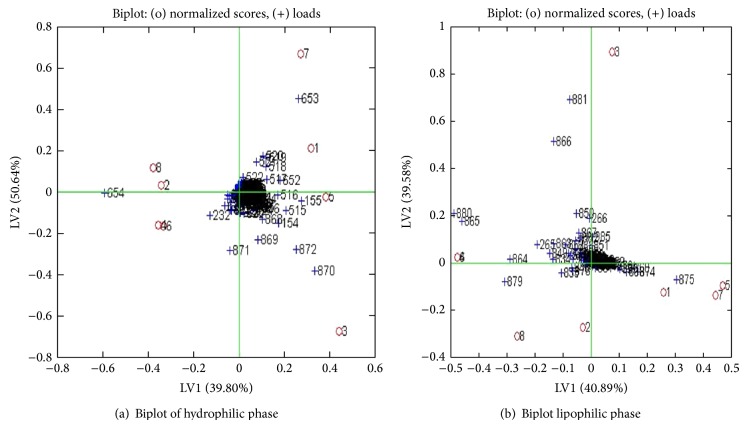
Biplot of OSC-PLS of control and ginger treated in both figures crosses indicates metabolites and circles indicate samples. Odd number circles are ginger treated and even number circles are controls. The crosses which are outliers correspond to the differentiating metabolites.

Figures 4(a) and 4(b) show score plot OSC-PLS modeling for the two groups of control and drug treated in both hydrophilic and lipophilic extracts. Odd numbers indicate control group and even numbers are related to treated extract (treated with drug). A good separation is seen between the two groups in the two phases. The biplots of OSC-PLS with application for the two groups are shown in Figures 5(a) and 5(b).

The numbers of the altered metabolites from the figures correlated with the chemical shifts in the spectra in the above graphs are in fact the entries of altered metabolites. Using the reference databank of HMDB the metabolites were identified. After processing the samples, the hydrophilic and lipophilic metabolites were obtained as shown in Table 1.

**Figure 6:**
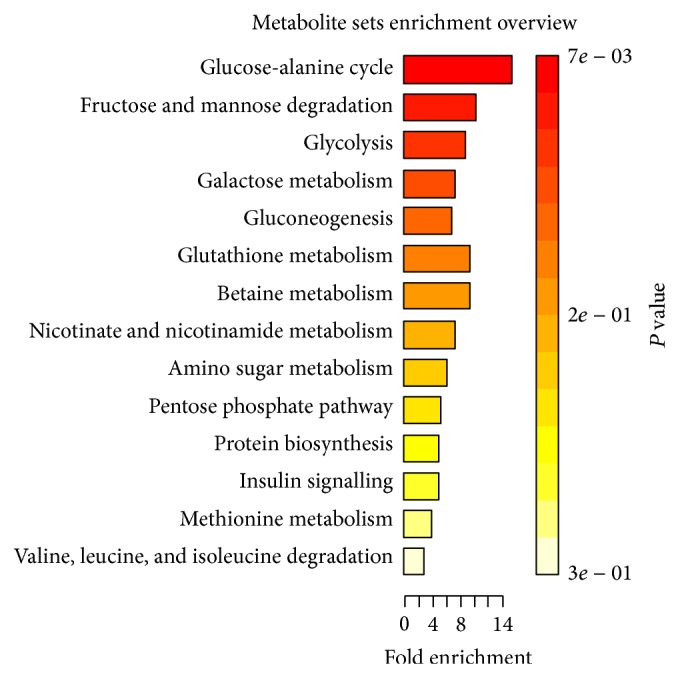
Enrichment analysis indicating important metabolic pathways in the hydrophilic phase *P* values in descending order.

**Figure 7:**
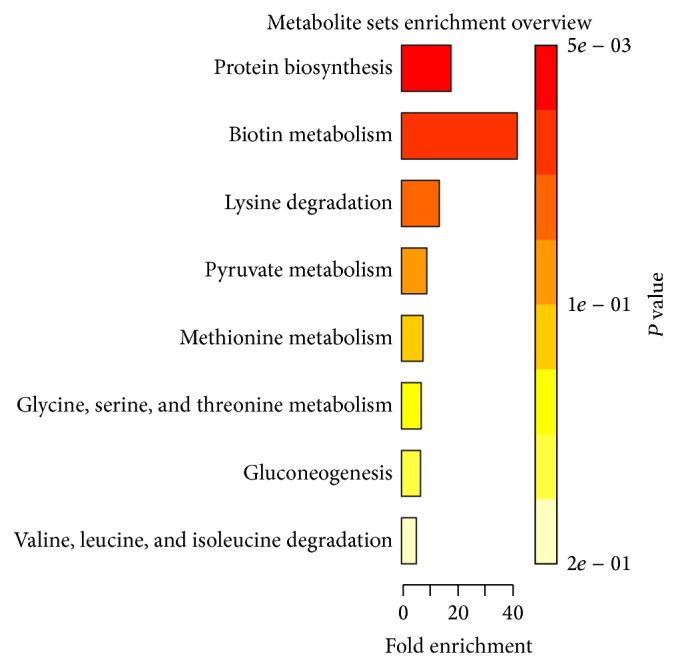
Enrichment analysis indicating important metabolic pathways in lipophilic phase *P* values in descending order.

For identification of the affected pathways, the detected metabolites were inserted in the upload option of the MetaboAnalyst software. The affected metabolic pathways of the two phases are seen in Figures 6 and 7; the *P* values obtained using enrichment analysis are in descending order (Tables 2 and 3).

The two different extracts obtained separated the metabolites into two groups: the lipophilic phase consisted mainly of amino acids and their metabolic cycles and the hydrophilic phase chiefly detected glucose cycles and their cycles. However, as both the phases are from the same cells, it can be concluded that ginger affects both amino acids and carbohydrate metabolism.

## 5. Discussion

The plants of the ginger family are widely included in diets throughout the world. The oleoresin extracted from the roots of ginger contains gingerol, which is a pharmacologically active substance [17, 18]. The impact of these substances on proliferation inhibition of human cancer cells through apoptosis pathway has been demonstrated [19]. Many herbs and spices have pharmacological and biochemical properties, including antioxidant and anti-inflammatory effects, which appear to be involved in anticancer and antimutagenic activities in a cell [20]. More than 50 antioxidants have been isolated from the ginger rhizome [21]. The most important antioxidant of ginger is [6]-gingerol, which has a sharp taste with significant antioxidant properties. Stimulation of the inhibitory phospholipid peroxidation in FeCl ascorbate system has been demonstrated [22]. 6-Gingerol has an inhibitory effect on the xanthine oxidase system [23] which is responsible for production of reactive oxygen species such as superoxide anion. In another study, the inhibitory effect of gingerol on arachidonic acids causing platelet aggregation and formation of thromboxane B2 and prostaglandin D2 has been demonstrated [24]. Gingerol, shogaol, and similar components in ginger inhibit the biosynthesis of leukotrienes and prostaglandins by inhibiting 5-lipooxygenase and prostaglandin synthetase pathways [25].

Studies have shown that 6-gingerol and 6-shogaol are responsible for therapeutic activities including antipyretic, analgesic, antitussive, and antihypertensive effects. In addition, they have antioxidant, anticancer, anti-inflammatory, antiangiogenesis, and antiarteriosclerotic properties [26, 27]. Notably, these compounds cause a low expression level of the gene regulating NF-*κ*B (nuclear factor kappa-light-chain-enhancer of activated B cells) which is a protein complex that controls transcription of DNA and is involved in cell proliferation and angiogenesis, resulting in apoptosis induction. In addition, they lower the level of IL-8 (interleukin-8) and VEGF 21 (vascular endothelial growth factor 21). Ginger extract inhibits platelet aggregation and thromboxane synthesis* in vitro*. This causes concerns about prolonged bleeding, but several European studies indicate that oral consumption of ginger has no significant anticoagulant effect [28].

Earlier studies carried out by us have shown that methanolic ginger extracts affect the sialic acid bound to O linked glycoproteins and mannose binding glycoproteins in Raji cells [29]. This study has shown the inhibitory effect of methanolic ginger extract on Raji cells, as proved by MTT and cell counting assay. The main metabolic cycles with the best *P* values which have changed in the Raji cells due to methanolic ginger extract are as follows: protein biosynthesis, glucose-alanine cycle, fructose and mannose degradation, glycolysis, and biotin metabolism.

### 5.1. Protein Biosynthesis

Involving the metabolites of L-lysine and L-isoleucine, these metabolites have been previously reported to play an important role in different cancers. Studies have shown that L-leucine or L-isoleucine supplementation enhanced growth of bladder urothelial tumors in rats by triggering expression of amino acid transporters and tumorigenesis-associated genes [30]. Methylation and acetylation of L-lysine seem to be important in promotion of proliferation of tumor cells. A report states that enhanced lysine methylation of HSP70 promoted proliferation of cancer cells through activation of a kinase enzyme named aurora kinase B [31]. Another study has shown that lysine acetylation promotes tumor growth through activation of 6-phosphogluconate dehydrogenase [32].

Lysine is also seen to participate in biotin metabolism [33]. Biotin uptake is described in small cell lung cancer cells, and it is seen that expression of oncogenes depends on it. Biotin uptake by breast cancer cells is higher as compared to normal cells for maintaining their high proliferative status [34]. This status is maintained by activation of oncogenes and loss of tumor suppressors which change metabolism and induce aerobic glycolysis. NADPH detected in our study acts along with glutamine, glucose, and ATP via the glycolysis pathway to provide the carbon skeletons to build new cancer cells [35].

As mentioned earlier, the effect of gingerol and shogaol on lowering the expression of the gene regulating protein biosynthesis such as NF-*κ*B has been described. In recent years, the focus of cancer research is slowly shifting to the study of alteration of protein production, cell proliferation, cell volume, and/or biomarkers of protein synthesis which may help predict response to drugs targeting cancer metabolism [36].

Fructose and mannose degradation in which fructose-6-phosphate and D-glucose were detected was seen in our enrichment analysis. Fructose is reported to be significant in breast cancers and renal cell carcinomas [37, 38]. Ginger has an effect on these pathways in Raji cells.

The next important carbohydrate pathways are the glucose-alanine pathway and glycolysis which were marked by D-glucose and NADP and D-glucose and fructose-6-phosphate, respectively. As mentioned above, bioenergetics of cancer cells have shown that glucose is the main energy source for body cells and tumor cells consume a high level of glucose [39]. Changing glucose metabolism is one of the hallmarks of cancer. In 1929, Otto Warburg was the first to demonstrate that, unlike somatic cells, cancer cells use glucose in the anaerobic pathway, and there is an increase in lactate production rather than oxidative phosphorylation and ATP production. As a result, the pH of the surrounding tissues is decreased, further damaging DNA repair mechanisms [40]. Therefore, lactate which is detected in Table 1 is reported as a key intermediate in tumor metabolism, and most tumor cells use lactate for energy consumption. Tumor cells have a high glycolytic activity due to increased glycolysis [41]. In the later stages, the lactate produced is delivered to the liver and muscles and is converted to glycogen and intermediate metabolites of Krebs cycle are reduced [42]. The level of glucose is reduced because of its high consumption. There is increased turnover and activity of membrane phospholipids. According to other studies, there is an increase in the level of pyruvate and lactate in sera of cancer patients due to high consumption of energy and glycolysis. Ginger affects the glycolytic cycle as shown in our studies, and reports have shown its effect on activity of glycolytic enzymes in diabetic patients [43].

## 6. Conclusion

Methanolic ginger extract has an inhibitory effect on Raji cells even at low concentrations of 1000 *μ*g/mL. Of the metabolites detected, lactate is reported as the key in tumor metabolism. Of the different cycles, proteins and carbohydrates metabolism were affected. In fact, the cycles detected by our metabonomic study have been reported earlier in genetics and immunology studies of ginger.

## Figures and Tables

**Table 1:** List of differentiating hydrophilic and lipophilic metabolites.

Number in the biplot	Name of metabolite	Chemical shift and multiplicity^a^
Lipophilic metabolites		
880	2-Ketobuytric acid	1.07 (t)
875	Isobutyryl-L-carnitine	1.14 (dd)
865	Isoleucine	1.24 (m)
895	Lactate	1.31 (d)
850	Homo-L-arginine	1.40 (m)
835	Lysine	1.54 (m)

Hydrophilic metabolites		
870	Isobutyryl-L-carnitine	1.14 (dd)
868	Isoleucine	1.24 (m)
528	Homo-L-arginine	1.63 (m)
653	D-Mannose	3.37 (dd)
624	Fructose-6-phosphate	3.64 (m)
526	Glucose	4.67 (d)
515	S-Adenosylhomocysteine	4.72 (t)
232	5-Methylcytidine	7.68 (s)
155	NADP	8.49 (s)

^a^s: single, d: doublet, t: triplet, q: quartet, m: multiplet, and dd: double doublet.

**Table 2:** Overrepresentation analysis of hydrophilic phase.

?	Total	Expected	Hits	Raw *P* value
Glucose-alanine cycle	12	0.13	2	6.60*E* − 01
Fructose and mannose degradation	18	0.20	2	1.48*E* − 02
Glycolysis	21	0.23	2	2.00*E* − 02
Galactose metabolism	25	0.27	2	2.79*E* − 02
Gluconeogenesis	27	0.29	2	3.22*E* − 02
Glutathione metabolism	10	0.11	1	1.04*E* − 01
Betaine metabolism	10	0.11	1	1.04*E* − 01
Nicotinate and nicotinamide metabolism	13	0.14	1	1.34*E* − 01
Amino sugar metabolism	15	0.16	1	1.53*E* − 01
Pentose phosphate pathway	18	0.20	1	1.81*E* − 01
Protein biosynthesis	19	0.21	1	1.90*E* − 01
Insulin signalling	19	0.21	1	1.90*E* − 01
Methionine metabolism	24	0.26	1	2.34*E* − 01
Valine, leucine, and isoleucine degradation	36	0.39	1	3.32*E* − 01

**Table 3:** Overrepresentation analysis of lipophilic phase.

	Total	Expected	Hits	Raw *P* value
Protein biosynthesis	19	0.12	2	4.83*E* − 03
Biotin metabolism	4	0.02	1	2.41*E* − 02
Lysine degradation	13	0.08	1	7.65*E* − 02
Pyruvate metabolism	20	0.12	1	1.16*E* − 01
Methionine metabolism	24	0.14	1	1.38*E* − 01
Glycine, serine, and threonine metabolism	26	0.16	1	1.48*E* − 01
Gluconeogenesis	27	0.16	1	1.54*E* − 01
Valine, leucine, and isoleucine degradation	36	0.22	1	2.00*E* − 01

## References

[1] International Agency for Research on Cancer http://www.uicc.org/resources/globocan.

[2] American Cancer Society (2011). *Cancer Facts & Figures 2011*.

[3] Remesh A. (2012). Toxicities of anticancer drugs and its management. *International Journal of Basic & Clinical Pharmacology*.

[4] Holohan C., van Schaeybroeck S., Longley D. B., Johnston P. G. (2013). Cancer drug resistance: an evolving paradigm. *Nature Reviews Cancer*.

[5] Cragg G. M., Newman D. J. (2005). Plants as a source of anti-cancer agents. *Journal of Ethnopharmacology*.

[6] Castleman M. (2001). *The New Healing Herbs*.

[7] Antoine A. A Current Look at Ginger Use.

[8] Ozgoli G., Goli M., Simbar M. (2009). Effects of ginger capsules on pregnancy, nausea, and vomiting. *Journal of Alternative and Complementary Medicine*.

[9] Park Y. J., Wen J., Bang S., Park S. W., Song S. Y. (2006). [6]-Gingerol induces cell cycle arrest and cell death of mutant p53-expressing pancreatic cancer cells. *Yonsei Medical Journal*.

[10] Bordia A., Verma S. K., Srivastava K. C. (1997). Effect of ginger (*Zingiber officinale* Rosc.) and fenugreek (*Trigonella foenumgraecum* L.) on blood lipids, blood sugar and platelet aggregation in patients with coronary artery disease. *Prostaglandins, Leukotrienes & Essential Fatty Acids*.

[11] Lindon J. C., Holmes E., Bollard M. E., Stanley E. G., Nicholson J. K. (2004). Metabonomics technologies and their applications in physiological monitoring, drug safety assessment and disease diagnosis. *Biomarkers*.

[12] Karpova M. B., Schoumans J., Ernberg J., Henter J.-I., Nordenskjöld M., Fadeel B. (2005). Raji revisited: cytogenetics of the original Burkitt's lymphoma cell line. *Leukemia*.

[13] Strobe W. (2001). Trepan blue exclusion test of cell viability. *Current Protocols in Immunology*.

[14] Scudiero D. A., Shoemaker R. H., Paull K. D., Monks A., Tierney S., Nofziger T. H., Currens M. J., Seniff D., Boyd M. R. (1988). Evaluation of a soluble tetrazolium/formazan assay for cell growth and drug sensitivity in culture using human and other tumor cell lines. *Cancer Research*.

[15] Gottschalk M., Ivanova G., Collins D. M., Eustace A., O'Connor R., Brougham D. F. (2008). Metabolomic studies of human lung carcinoma cell lines using *in vitro*
^1^H NMR of whole cells and cellular extracts. *NMR in Biomedicine*.

[16] Wold H. (1966). *Research Papers in Statistics*.

[17] Singh A., Duggal S., Singh J., Katekhaye S. (2010). Experimental advances in pharmacology of gingerol and analogues. *Pharmacy Global: International Journal of Comprehensive Pharmacy*.

[18] Suekawa M., Ishige A., Yuasa K., Sudo K., Aburada M., Hosoya E. (1984). Pharmacological studies on ginger. I. Pharmacological actions of pungent constituents, (6)-gingerol and (6)-shogaol. *Journal of Pharmacobio-Dynamics*.

[19] Chakraborty D., Bishayee K., Ghosh S., Biswas R., Mandal S. K., Khuda-Bukhsh A. R. (2012). [6]-Gingerol induces caspase 3 dependent apoptosis and autophagy in cancer cells: drug-DNA interaction and expression of certain signal genes in HeLa cells. *European Journal of Pharmacology*.

[20] Lin R.-J., Chen C.-Y., Chung L.-Y., Yen C.-M. (2010). Larvicidal activities of ginger (*Zingiber officinale*) against *Angiostrongylus cantonensis*. *Acta Tropica*.

[21] Chang W.-S., Chang Y.-H., Lu F.-J., Chiang H.-C. (1994). Inhibitory effects of phenolics on xanthine oxidase. *Anticancer Research*.

[22] Yusof Y. A. M., Ahmad N., Das S., Sulaiman S., Murad N. A. (2009). Chemopreventive efficacy of ginger (*Zingiber officinale*) in ethionine induced rat hepatocarcinogenesis. *African Journal of Traditional, Complementary and Alternative Medicines*.

[23] Kiuchi F., Shibuya M., Sankawa U. (1982). Inhibitors of prostaglandin biosynthesis from ginger. *Chemical and Pharmaceutical Bulletin*.

[24] Guh J.-H., Ko F.-N., Jong T.-T., Teng C.-M. (1995). Antiplatelet effect of gingerol isolated from Zingiber officinale. *Journal of Pharmacy and Pharmacology*.

[25] Flynn D. L., Rafferty M. F., Boctor A. M. (1986). Inhibition of human neutrophil 5-lipoxygenase activity by gingerdione, shogaol, capsaicin and related pungent compounds. *Prostaglandins, Leukotrienes and Medicine*.

[26] Singh A., Duggal S., Singh J., Katekhaye Sh. (2010). Experimental advances in pharmacology of gingerol and analogues. *Pharmacie Globale: International Journal of Comprehensive Pharmacy*.

[27] Shoji N., Iwasa A., Takemoto T., Ishida Y., Ohizumi Y. (1982). Cardiotonic principles of ginger (Zingiber officinale Roscoe). *Journal of Pharmaceutical Sciences*.

[28] Janssen P., Meyboom S., Staveren W. (1996). Consumption of ginger (Zingiber Officinale Roscoe) does not affect ex vivo platelet thromboxane production in humans. *European Journal of Clinical Nutrition*.

[29] Zamani Z., Kohan H. K., Kadivar M., Kalyee Z., Rad Laame B., Iravani A., Rahimi N. A., Wahabi F., Sadeghi S., Pourfallah F., Arjmand M. (2014). The effect of ginger extract on glycoproteins of Raji cells. *Pakistan Journal of Biological Sciences*.

[30] Xie X., Kakehashi A., Wei M., Yamano S., Takeshita M., Yunoki T., Wanibuchi H. (2013). L-Leucine and l-isoleucine enhance growth of BBN-induced urothelial tumors in the rat bladder by modulating expression of amino acid transporters and tumorigenesis-associated genes. *Food and Chemical Toxicology*.

[31] Cho H.-S., Shimazu T., Toyokawa G., Daigo Y., Maehara Y., Hayami S., Ito A., Masuda K., Ikawa N., Field H. I., Tsuchiya E., Ohnuma S.-I., Ponder B. A. J., Yoshida M., Nakamura Y., Hamamoto R. (2012). Enhanced HSP70 lysine methylation promotes proliferation of cancer cells through activation of Aurora kinase B. *Nature Communications*.

[32] Shan C., Elf S., Ji Q. (2014). Lysine acetylation activates 6-phosphogluconate dehydrogenase to promote tumor growth. *Molecular Cell*.

[33] Vadlapudi A. D., Vadlapatla R. K., Pal D., Mitra A. K. (2013). Biotin uptake by T47D breast cancer cells: functional and molecular evidence of sodium-dependent multivitamin transporter (SMVT). *International Journal of Pharmaceutics*.

[34] Scheerger S. B., Zempleni J. (2003). Expression of oncogenes depends on biotin in human small cell lung cancer cells NCI-H69. *International Journal for Vitamin and Nutrition Research*.

[35] Dang C. V. (2012). Links between metabolism and cancer. *Genes and Development*.

[36] Dolfi S., Chan L. L.-Y., Qiu J., Tedeschi P. M., Bertino J. R., Hirshfield K. M., Oltvai Z. N., Vazquez A. (2009). The metabolic demands of cancer cells are coupled to their size and protein synthesis rates. *Molecular Oncology*.

[37] Zaravinos A., Pieri M., Mourmouras N., Anastasiadou N., Zouvani I., Delakas D., Deltas C. (2014). Altered metabolic pathways in clear cell renal cell carcinoma: a meta-analysis and validation study focused on the deregulated genes and their associated networks. *Oncoscience*.

[38] Schramm G., Surmann E.-M., Wiesberg S., Oswald M., Reinelt G., Eils R., König R. (2010). Analyzing the regulation of metabolic pathways in human breast cancer. *BMC Medical Genomics*.

[39] Huang X., Chen Q., Yang G., Dai W., Lang Q., Du J., Yan S., Zhang W., Ling C. (2012). Metabolic profiling study of yang deficiency syndrome in hepatocellular carcinoma by ^1^H NMR and pattern recognition. *Evidence-based Complementary and Alternative Medicine*.

[40] Fong M. Y., McDunn J., Kakar S. S. (2011). Identification of metabolites in the normal ovary and their transformation in primary and metastatic ovarian cancer. *PLoS ONE*.

[41] Serkova N. J., Spratlin J. L., Eckhardt S. G. (2007). NMR-based metabolomics: translational application and treatment of cancer. *Current Opinion in Molecular Therapeutics*.

[42] Slupsky C. M., Steed H., Wells T. H., Dabbs K., Schepansky A., Capstick V., Faught W., Sawyer M. B. (2010). Urine metabolite analysis offers potential early diagnosis of ovarian and breast cancers. *Clinical Cancer Research*.

[43] Abdulrazaq N. B., Cho M. M., Win N. N., Zaman R., Rahman M. T. (2012). Beneficial effects of ginger (Zingiber officinale) on carbohydrate metabolism in streptozotocin-induced diabetic rats. *British Journal of Nutrition*.

